# Theta and beta synchrony coordinate frontal eye fields and anterior cingulate cortex during sensorimotor mapping

**DOI:** 10.1038/ncomms13967

**Published:** 2017-02-07

**Authors:** Sahand Babapoor-Farrokhran, Martin Vinck, Thilo Womelsdorf, Stefan Everling

**Affiliations:** 1 Neuroscience Graduate Program, University of Western Ontario, London, Ontario, Canada N6A 5K8; 2 Department of Psychiatry, Schulich School of Medicine and Dentistry, University of Western Ontario, London, Ontario, Canada N6A 5W9; 3 Department of Neurobiology, School of Medicine, Yale University, New Haven, Conneticut 06520, USA; 4 Ernst Strüngmann Institut (ESI) for Neuroscience in Cooperation with Max Planck Society, Frankfurt 60528, Germany; 5 Department of Biology, Centre for Vision Research, York University, Toronto, Ontario, Canada M3J 1P3; 6 Robarts Research Institute, London, Ontario, Canada N6A 5K8; 7 Department of Physiology and Pharmacology, University of Western Ontario, London, Ontario, Canada N6A 5C1

## Abstract

The frontal eye fields (FEFs) and the anterior cingulate cortex (ACC) are commonly coactivated for cognitive saccade tasks, but whether this joined activation indexes coordinated activity underlying successful guidance of sensorimotor mapping is unknown. Here we test whether ACC and FEF circuits coordinate through phase synchronization of local field potential and neural spiking activity in macaque monkeys performing memory-guided and pro- and anti-saccades. We find that FEF and ACC showed prominent synchronization at a 3–9 Hz theta and a 12–30 Hz beta frequency band during the delay and preparation periods with a strong Granger-causal influence from ACC to FEF. The strength of theta- and beta-band coherence between ACC and FEF but not variations in power predict correct task performance. Taken together, the results support a role of ACC in cognitive control of frontoparietal networks and suggest that narrow-band theta and to some extent beta rhythmic activity indexes the coordination of relevant information during periods of enhanced control demands.

## Abstract (web summary)

Frontal eye fields (FEF) and anterior cingulate cortex (ACC) are coactivated during cognitive tasks, but the precise format of their interaction is not known. Here the authors show that phase coupling between ACC -FEF in theta and beta frequency bands better predicts behavioural performance.

The frontal eye field (FEF) plays a crucial role in overt^1^,^2^ and covert orienting^3^,^4^. Together with parietal and extrastriate visual areas, FEF is part of the dorsal attention network that provides top-down allocation of attention to contralateral space through long-range connections with visual cortical areas in humans and non-human primates^5^,^6^. Activity in this frontoparietal spatial priority network is modulated by cortical and subcortical inputs that are thought to encode behavioural rules, values, and motivational signals^7^. One cortical area that has anatomical connections with FEF is the anterior cingulate cortex (ACC)^8^. Functional connectivity between ACC and FEF has also been demonstrated by resting-state functional magnetic resonance imaging (fMRI) in macaques^9^, and task-based fMRI studies commonly show coactivation of FEF and ACC in a variety of cognitive saccades task^10^. Furthermore, a recent task base functional connectivity study in humans suggested that ACC might act as a high-level motor control region^11^. However, the neural mechanisms underlying the functional coactivation of ACC and FEF in behavioural tasks remain unknown.

One proposed mechanism for the communication of spatially dispersed neuronal groups is through synchronizing the excitability phases of band-limited activity^12^,^13^. Such long-range phase synchrony of rhythmic activation facilitates the efficient transmission of neuronal signals between brain regions^12^,^14^. More specifically, the communication between brain areas involved in memory-guided and top-down control processes is thought to be facilitated in prefrontal cortices via synchronization at band-limited rhythmic activity in a theta frequency band (3–9 Hz)^15^,^16^ and in a beta frequency band (12–30 Hz)^17^,^18^,^19^,^20^,^21^. However, the contribution of theta and beta frequency bands to the transmission of neural signals between ACC and other brain areas is still poorly understood.

Here we recorded local field potentials (LFPs) and spiking activity from FEF and ACC simultaneously and investigated task-dependent synchronization between the two areas while monkeys performed a memory-guided saccade task and a task with randomly interleaved pro- and anti-saccade trials. The results show that a narrow-band theta and beta rhythmic activity indexes the coordination of relevant information between FEF and ACC during periods of enhanced control demands.

## Results

### Recording specifications

**Figure 1:**
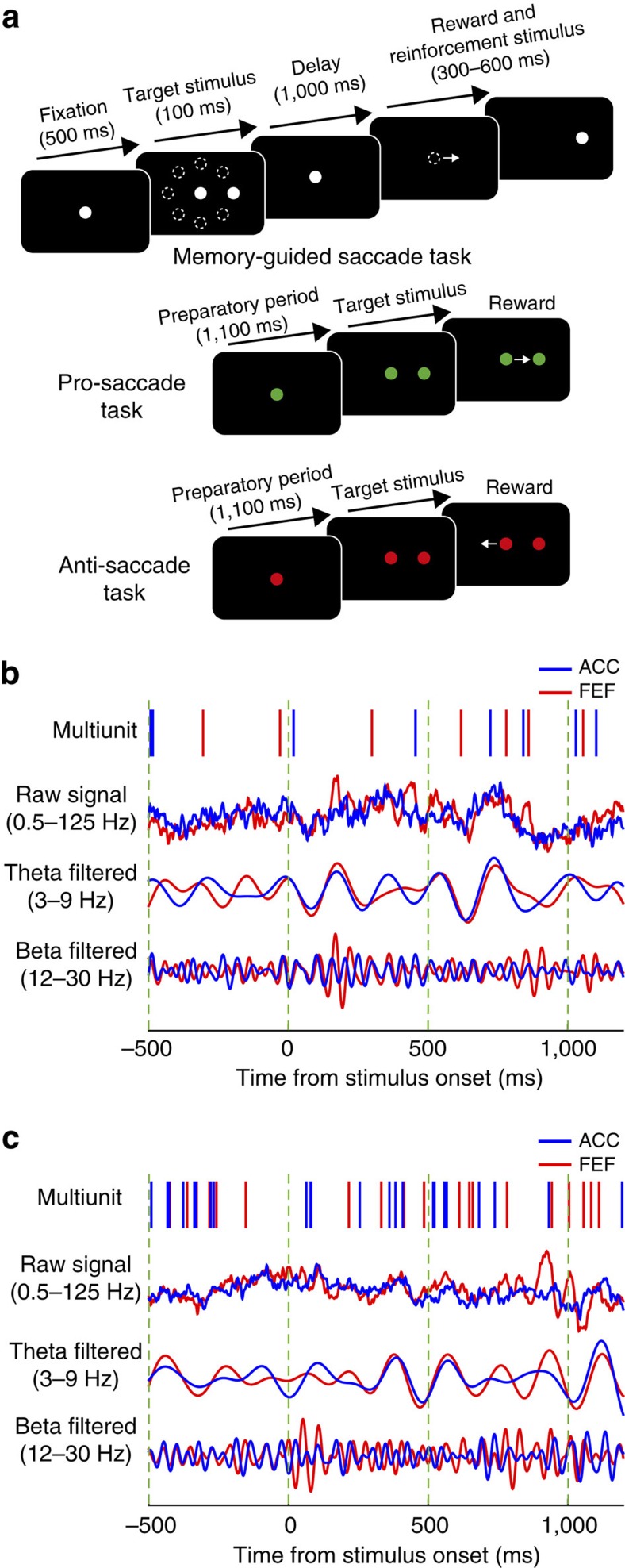
Experimental paradigm and sample traces of simultaneously recorded activity in ACC and FEF. (**a**) Schematic of the memory-guided saccade task and pro-/anti-saccade task. (**b**) The traces show the multiunit activity, raw LFP signal (0.5–125 Hz), theta band-pass-filtered signal (3–9 Hz), and beta band-pass-filtered signal (12–30 Hz) in a trial of memory-guided saccade task. (**c**) Same as in **a** in another memory-guided saccade task trial. The vertical green dashed lines indicate the 500 ms time intervals aligned on stimulus onset.

We simultaneously recorded LFPs in the ACC (*n*=233 sites; *n*=87 and *n*=146 in monkey 1 and 2, respectively) and FEF (*n*=209 sites; *n*=62 and *n*=147 in monkey 1 and 2) while monkeys performed the memory-guided saccade task and the pro-/anti-saccade task (Fig. 1a). Together with these LFPs, we simultaneously recorded the spiking activity of ACC (*n*=149 units; *n*=52 and *n*=97 in monkey 1 and 2) and FEF units (*n*=74 units; *n*=46 and *n*=28 in monkey 1 and 2; Fig. 1b,c).

**Figure 2:**
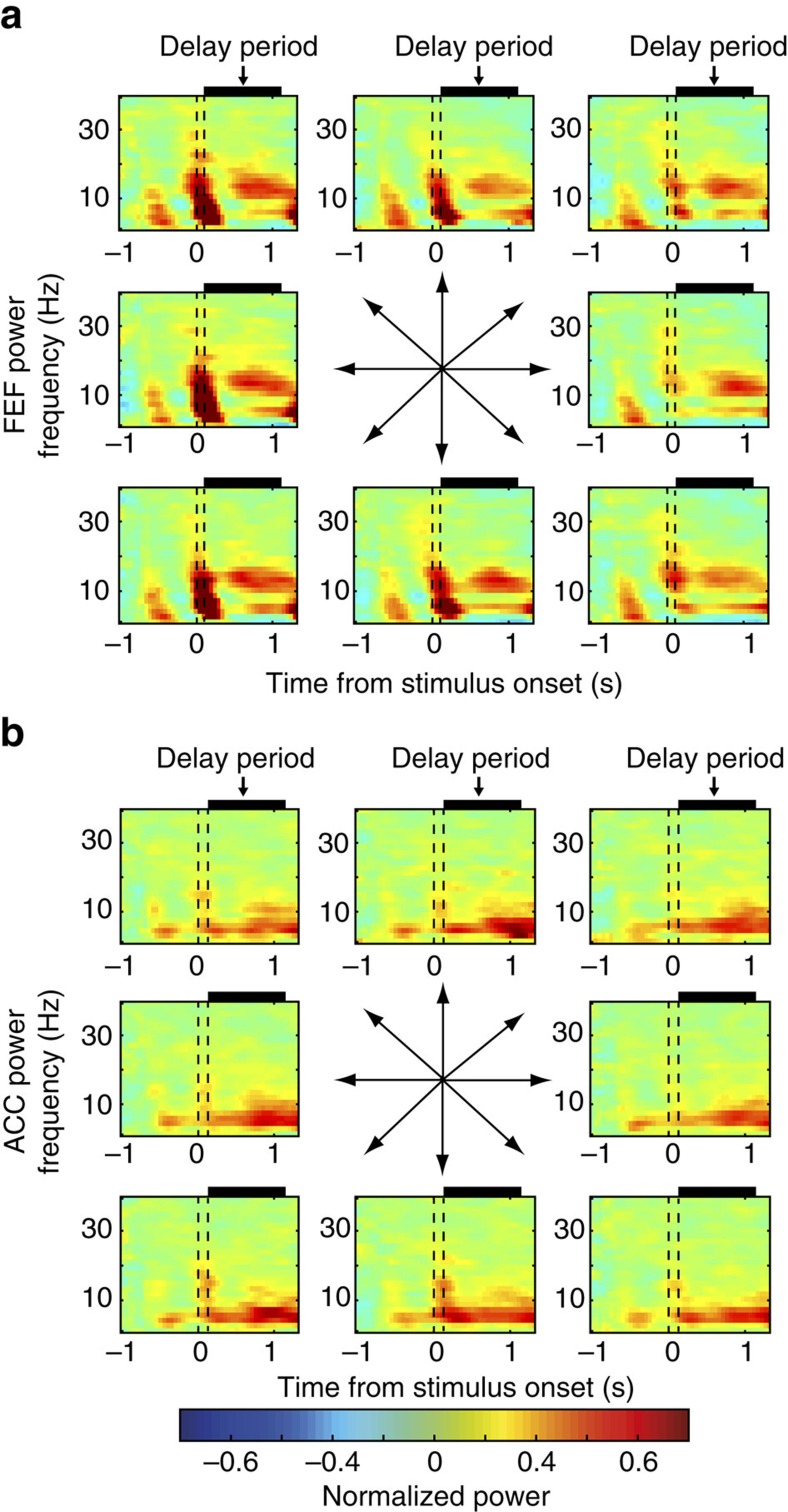
LFP power in FEF and ACC during the memory-guided saccade task. (**a**) Average time-frequency spectra of the FEF LFP power across eight target locations in the memory-guided saccade task. (**b**) Average time frequency spectra of ACC LFP power across eight target locations in the memory-guided saccade task. The dashed lines demarcate the time of the onset and offset of the target stimulus. The black boxes on top of each graph demarcate the delay period.

**Figure 3:**
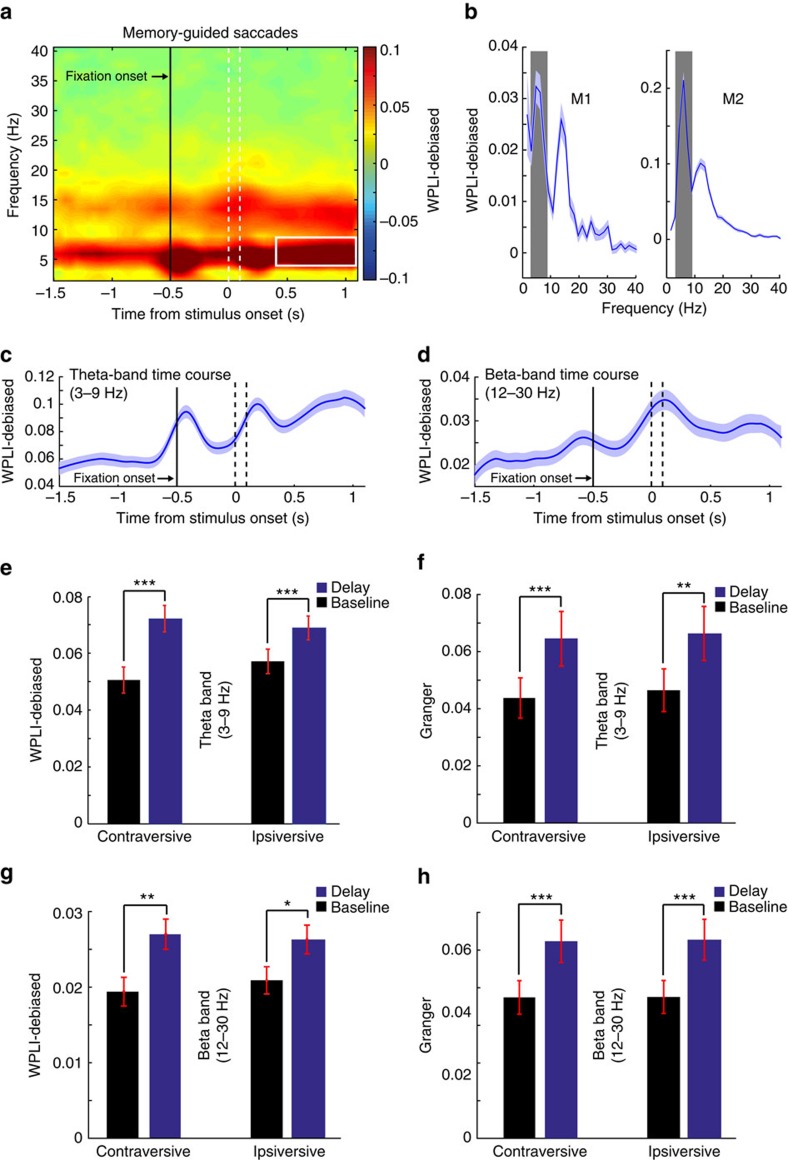
Increased theta and beta coherence between ACC and FEF. (**a**) Time-frequency spectrum of the WPLI-debiased coherence between the FEF and ACC in memory-guided saccade task for the population of ACC-FEF channel pairs (*n*=674). The white contour shows the area in which the subsequent analyses were performed (see Methods). The dashed lines demarcate the time of the onset and offset of the target stimulus. (**b**) WPLI-debiased FEF-ACC coherence spectrum of the individual monkeys in the delay period across all recording pairs (*n*=674). (**c**) Theta-band (3–9 Hz) time course of the ACC-FEF WPLI-debiased phase synchronization. (**d**) Beta-band (12–30 Hz) time course of the ACC-FEF WPLI-debiased phase synchronization. (**e**) Comparison of WPLI-debiased coherence between baseline and delay period of the contra- and ipsiversive memory-guided saccades (***P<0.001, *t*-test). Error bars indicate s.e.m. (**f**) Comparison of the overall Granger-causality influence of ACC over FEF (GACC→FEF−GFEF→ACC) between baseline and delay period of the contra- and ipsiversive memory-guided saccades (***P<0.001, **P<0.01, *t*-test, *n*=275). Error bars indicate s.e.m. (**g**) Comparison of beta-band WPLI-debiased coherence between baseline and delay period of the contra- and ipsiversive memory-guided saccades (**P<0.01, *P<0.05; *t*-test). Error bars indicate s.e.m. (**h**) Comparison of the beta-band overall Granger influence of ACC over FEF (GACC→FEF−GFEF→ACC) between baseline and delay period of the contra- and ipsiversive memory-guided saccades (***P<0.001; *t*-test). Error bars indicate s.e.m.

LFP power and phase-synchronization spectra showed prominent peaks in the theta and beta band (Figs 2 and 3). We therefore focus the remainder of the results and discussion on these two bands. We also explored ACC-FEF interactions across delta, alpha, and gamma (30–100 Hz) bands. In contrast to the theta and beta band, we observed no consistent modulation in these frequency ranges in different conditions (Figs 2 and 3 and Supplementary Figs 2–7), suggesting that functionally meaningful interactions between ACC and FEF proceed predominantly in theta and beta frequencies^7^.

### Modulation of theta and beta power during the delay period

The memory-guided saccade task required the monkeys to maintain a spatial location in working memory and then to generate a saccade toward the remembered location after the imposed delay period. Note that since in this task spatial information may be transformed into saccade information before the delay period, there is a possibility that the monkeys hold the saccade information in working memory. We first calculated the LFP power spectra during the delay period for correctly performed memory-guided saccade trials (horizontal and oblique targets). Both ACC and FEF LFPs showed a prominent increase in theta power (3–9 Hz) during the delay period (Fig. 2a,b). Additionally, a transient increase in 15–20 Hz beta frequency power was evident in the immediate poststimulus period (Fig. 2a,b).

We further examined whether LFP power was tuned to the spatial target location. We found that a significant number of FEF (18/209; 8.61%, *P*<0.05, Binomial test) but not ACC (11/233; 4.72% NS; ACC versus FEF: *P*=0.099, Chi-square test) channels showed significant spatial tuning of power within the theta frequency band (3–9 Hz) during the delay period in the time window of 400–1,100 ms following target stimulus onset (Fig. 2a, one-way analysis of variance, *P*<0.05). In addition, a significant number of FEF (50/209; 23.92%, *P*<0.05, Binomial test) but not ACC channels (14/233; 6.08% NS; difference ACC versus FEF: *P*<0.001, Chi-square test) showed significant tuning in the beta band (12–30 Hz). Thus, we found spatial tuning of LFP power in theta and beta frequencies in FEF but not in ACC. The observed power modulation of the FEF LFPs is in line with previous FEF single-unit recording results, which suggest a high degree of spatial tuning in FEF neurons^22^.

### Task-dependent LFP-LFP coherence between ACC and FEF

We next tested whether ACC and FEF showed task-dependent interactions in the theta (3–9 Hz) and beta (12–30 Hz) band. For a large subset of interareal recording pairs, theta- and beta-band phase synchronization increased from the baseline (700 ms prior to the fixation point onset in the intertrial interval) to the delay period of the memory-guided saccade task (400 to 1,100 ms following stimulus onset in the delay period, see Methods). Of the 674 ACC-FEF LFP-LFP channel pairs, respectively 447 (66.32%) and 330 (48.96%) channel pairs displayed significant increases in theta- and beta-band phase synchronization during the delay period (theta: 141/233 and 306/441 of monkey 1 and 2, respectively; beta: 91/233 and 239/441 of monkey 1 and 2; *P*<0.001, permutation test, see Methods). On the other hand, respectively, 128 (18.99%) and 179 (26.56%) pairs exhibited statistically significant decreased theta and beta synchrony in the delay period relative to the baseline (theta: 65/233 and 63/441 of monkey 1 and 2; beta: 71/233 and 108/441 of monkey 1 and 2; *P*<0.001, permutation test). We observed that, respectively, 234/674 (34.72%), 213/674 (31.6%), and 96/674 (14.83%) of channel pairs exhibited increased phase synchronization across both theta and beta band, theta band only, and beta band only, during the delay period versus baseline. With regard to significant decreased phase synchrony in the delay period versus baseline, the following results were obtained: 39/674 (5.79%) channel pairs exhibited concurrent theta- and beta-band decrease, 89/674 (13.2%) pairs displayed theta- but not beta-band decrease, and 140/674 (20.77%) showed beta- but not theta-band phase synchrony decrease. Furthermore, the population of ACC-FEF channel pairs exhibited increased theta- and beta-band synchrony in the delay period versus baseline of the memory-guided saccade task (Fig. 3c–e,g). Consistent with these results, we found a prominent peak in the average interareal theta- and beta-, but not alpha-band (10–12 Hz) phase synchronization across the population of pairs of recording sites in FEF and ACC (Fig. 3a,b and Supplementary Fig. 2). This theta- and beta-band phase synchronization was evident in both monkeys (Fig. 3b). In addition, we observed increased interareal theta- and beta-band synchronization in the preparatory period (400–1,100 ms following fixation onset) of the pro-/anti-saccade task compared to baseline (Supplementary Fig. 2B).

Increased phase synchronization between ACC and FEF might partially reflect an independent phase reset in both areas aligned on stimulus onset. To examine this, we subtracted the averaged stimulus-aligned evoked LFP from the signals before calculating phase synchrony. This procedure resulted in decreased ACC-FEF theta-band phase synchronization during the early delay period (Fig. 3c compared to Supplementary Fig. 3C) but did not affect the increased phase synchronization in the late delay period (400–1,100 ms following the stimulus onset). This finding suggests that the increased phase synchronization in the 400–1,100 ms following the stimulus period is not time locked to the stimulus onset and reflects genuine ACC-FEF synchronization (Supplementary Fig. 3). On the other hand, we did not observe this decrease in the immediate poststimulus beta-band phase synchronization of the average-subtracted data compared to that of the original data (Fig. 3d compared to Supplementary Fig. 3D). This suggests that beta-band ACC-FEF phase synchronization primarily represents an induced oscillatory response that is not evoked by the stimulus onset. We also subtracted the evoked response by applying a different method described by Truccolo *et al*.^23^. The results of this analysis also confirmed that the observed theta- and beta-band phase synchronization in the 400–1,100 ms period following the stimulus onset is due to induced oscillatory activity in ACC and FEF (Supplementary Fig. 3E,F). In the following sections, we have performed the delay period analyses in the 400–1,100 ms following the stimulus onset to avoid stimulus-related evoked responses.

Finally, we asked whether interareal phase synchronization distinguished ipsi- and contraversive saccade planning in the memory-guided saccade task. There was no statistically significant difference between ipsi- and contraversive saccade conditions in phase synchronization across both theta and beta bands in the window of 400–1,100 ms following the stimulus onset (Fig. 3e,g).

To summarize, these findings indicate that there is a coordination of FEF and ACC activity in theta- and beta-band frequencies during the delay period of the memory-guided saccade task and the preparatory period of the pro-/anti-saccade task, which represent periods of enhanced control demands.

### Task-dependent Granger causality between ACC and FEF LFPs

We further investigated the directionality of the ACC-FEF interactions by performing Granger-causality analyses. Both during the delay and the baseline period, we observed bidirectional Granger-causal influences between ACC and FEF LFPs, with prominent peaks in the theta and beta range, and a significantly stronger influence from ACC to FEF than vice versa (*n*=275, paired *t*-test, *P*<0.05; Supplementary Fig. 4A–C). During the delay period, as compared to the baseline period, the Granger-causal influence in the theta band increased for both directions (GACC→FEF and GFEF→ACC) (Supplementary Fig. 4A,B). Yet, for the beta band, we observed that the Granger-causal influence increased from ACC to FEF, but not from FEF to ACC (Supplementary Fig. 4A,B). Correspondingly, we found that the Granger-causal influence in the theta band (*n*=275, paired *t*-test, *P*<0.001 in contraversive and *P*<0.01 in ipsiversive conditions; Fig. 3f) and beta band (*n*=275, paired *t*-test, *P*<0.001 in both contra- and ipsiversive trials; Fig. 3h) increased more strongly in the direction of ACC to FEF than vice versa (GACC→FEF−GFEF→ACC). The results also indicated that the influence of ACC on FEF is greater than that of FEF over ACC during the delay period across alpha and gamma frequency ranges (*n*=275, paired *t*-test, *P*<0.05), as shown in Supplementary Fig. 4C. We performed a similar bootstrapping test as described for the weighted phase lag index (WPLI)-debiased analysis, to investigate the significant difference between baseline and delay periods, across the single channel pairs with flipped Granger-causality sign following the reversal of time series. In the direction of GACC→FEF, respectively, 115/275 (41.82%), 47/275 (17.09%), and 41/275 (14.91%) of channel pairs exhibited concurrent theta and beta increase, theta-band-only increase, and beta-band-only increase in Granger causality in the delay period. On the other hand, respectively, 46/275 (16.73%), 34/275 (12.36%), and 52/275 (18.91%) of channel pairs displayed concurrent theta- and beta-band, theta-band-only, and beta-band-only decrease in Granger causality in the delay period. Within the direction of GFEF→ACC, we observed that, respectively, 100/275 (36.36%), 68/275 (24.73%), and 26/275 (9.45%) of channel pairs showed concurrent theta- and beta-band, theta-band-only, and beta-band-only increase in Granger causality in the delay period. Conversely, 51/275 (18.55%), 30/275 (10.91%), and 48/275 (17.45%) of channel pairs demonstrated decreased Granger causality concurrently across theta and beta band, across theta band only, and across beta band only, respectively.

We computed the chance level Granger values by randomly shuffling the time-frequency domain of the raw data. Across theta, alpha, beta, and gamma frequencies, the GACC→FEF and GFEF→ACC values, both in the baseline and delay periods, were above the chance level.

In summary, we observed that ACC-FEF interaction patterns changed during the delay period of the contra- and ipsiversive saccade trials, with the Granger-causal influence from ACC to FEF becoming relatively stronger.

### Decreased ACC-FEF theta and beta synchrony on error trials

**Figure 4:**
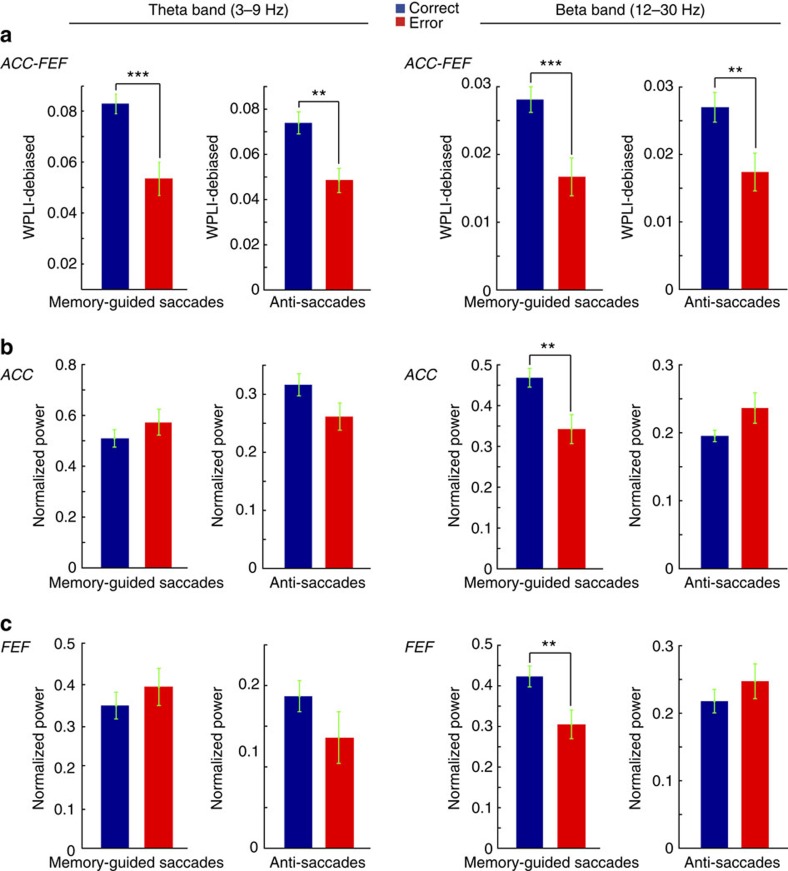
Correct task performance is dependent on field-field coherence but not on LFP power. (**a**) Shown are the comparison of theta- (columns 1 and 2) and beta-band (columns 3 and 4) WPLI-debiased coherence between correct and error memory-guided saccades in the delay period of the memory-guided saccade task (400–1,100 ms following target stimulus onset) and the preparatory period (400–1,100 ms following fixation onset) of the anti-saccade task. (**b**) Same as in **a**, but show normalized ACC theta- and beta-band power. (**c**) Same as in **a** but for normalized FEF power. *P<0.05; **P<0.01; ***P<0.001, *t*-test. Error bars indicate s.e.m.

We found that FEF and ACC LFPs exhibit phase synchronization in the theta and beta frequency bands during the delay period. Our next question was whether this synchronization predicted behavioural task performance. To test whether the strength of phase synchronization influenced performance in the memory-guided saccade task, we compared FEF-ACC phase synchronization between correct trials and error trials in which the animal made an eye movement toward the wrong stimulus location. We found that both theta- and beta-band coherence between the ACC and FEF were significantly larger on correct trials than error trials during the delay period (Fig. 4a; *n*=487, *t*-test, *P*<0.001). We validated the relationship of coherence with performance by using an anti-saccade task that triggered more directional error trials than the memory-guided saccade task. Similar to the memory-guided saccade task, the population of the ACC-FEF channel pairs showed significantly decreased theta- and beta-band phase synchronization in the error versus correct anti-saccades during the 400–1,100 ms following the fixation onset during the preparatory period (Fig. 4a, *n*=577, *t*-test, *P*<0.01). We confirmed that these statistical differences were not the result of differences in sample size, by performing the same analysis using the same number of trials for correct and error trials.

We also asked how theta power is modulated in correct and error trials in the ACC and FEF. We found no significant differences in theta power between correct and error trials in the ACC or FEF (Fig. 4b,c, left two columns). However, there were significant differences of beta power across the delay periods of the memory-guided saccade task but not in the preparatory period of the anti-saccade task (Fig. 4b,c, right two columns). This indicated that the changes in theta and beta band ACC-FEF phase synchronization in either one (beta) or both (theta) behavioural tasks were dissociated from changes in local power.

Taken together, these results indicate that theta- and beta-band phase synchronization between ACC and FEF provide a better prediction of performance than local power in these areas. Higher phase synchronization on correct trials than on error trials supports the hypothesis that ACC-FEF theta and beta phase synchronization play functional roles in working memory as well as in cognitive control.

### Modulation of spike-field synchrony during the delay period

Taken together, these results suggest that theta- and beta-band frequencies play a prominent role in the neuronal communication between ACC and FEF. We hence predicted that theta- and beta-band LFP modulations in one area should be related to the spiking activity of neurons in the other area. We tested this using the pairwise phase consistency (PPC) as a measure of spike-field coherence^24^. We first examined whether there was a difference in spike-field synchrony between the baseline and delay periods.

**Figure 5:**
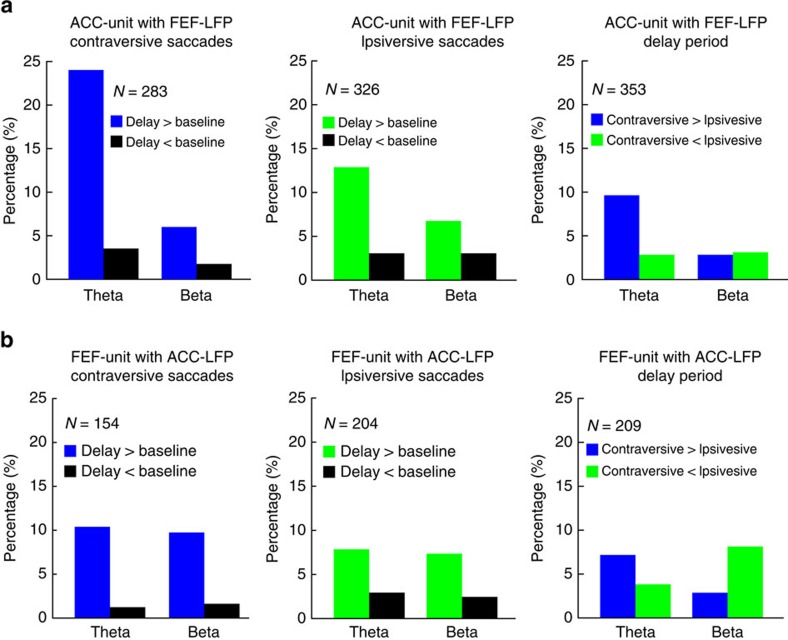
Percentage of units with significant spike-field coupling in theta and beta band. (**a**) Percentage of the ACC-unit with FEF-LFP pairs showing significant changes in phase locking across the theta and beta frequency range. Comparison between baseline and delay of contraversive saccades (left), comparison between baseline and delay of ipsiversive saccades (middle), and comparison between the contra- and ipsiversive saccades in the delay period (right). (**b**) Same as in **a**, but now depicted the percentage of the FEF-unit with ACC-LFP pairs showing significant changes in phase locking across the theta and beta frequency range. Statistical testing was performed using two-sided permutation tests, such that chance level is 2.5%.

**Figure 6:**
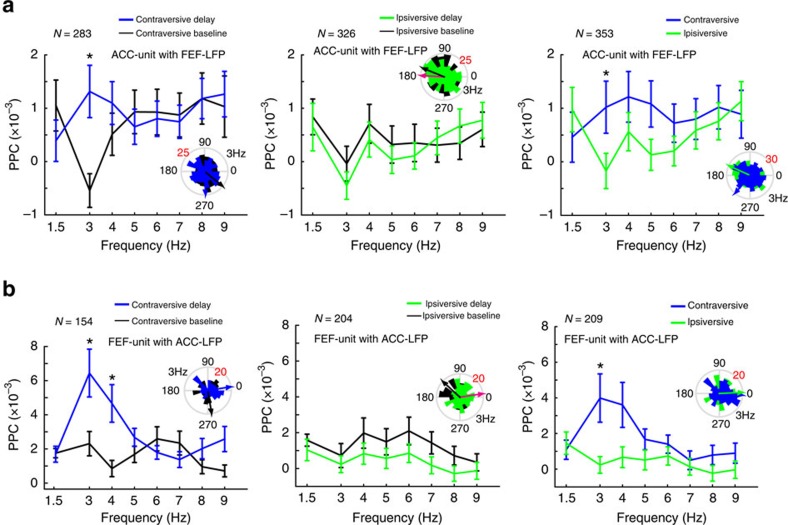
Pairwise phase consistencies (PPCs) across delta and theta band. (**a**) PPC spike-field coherence spectrum of the population of the ACC-unit with FEF-LFP pairs across the delta and theta frequency range. Comparison between baseline and delay of contraversive saccades (left), comparison between baseline and delay of ipsiversive saccades (middle), and comparison between the contra- and ipsiversive saccades in the delay period (right). (**b**) PPC spike-field coherence spectrum of the population of the FEF-unit with ACC-LFP pairs across the delta and theta frequency range. Comparison between baseline and delay of contraversive saccades (left), comparison between baseline and delay of ipsiversive saccades (middle), and comparison between the contra- and ipsiversive saccades in the delay period (right). It should be noted that the same significant differences between ipsi- and contraversive trials were seen even after we compared the contra- versus ipsiversive conditions using a permutation test as described in the Methods section. Error bars denote s.e.m. in all panels. *P<0.05, paired *t*-test. The rose plots on the side of each graph show the histogram of the coupling angles of the population of the ACC/FEF units.

A significant fraction of ACC-unit with FEF-LFP pairs and FEF-unit with ACC-LFP pairs exhibited significantly increased (permutation test; Fig. 5a,b, left and middle) theta-band phase coupling in the delay period versus baseline of both contraversive (ACC units: 68/283=24.03%, *P*<0.001; FEF units: 16/154=10.4%, *P*<0.001, Binomial test) and ipsiversive trials (ACC units: 45/326=13.8%, *P*<0.001; FEF units: 17/204=8.33%, *P*<0.001, Binomial test). In contrast, we found no evidence for significant decreases in phase locking (Fig. 5a,b, left and middle). Furthermore, we observed an increase in average spike-field coupling in a subset of theta frequencies (3–4 Hz) during delay versus baseline of contraversive, but not ipsiversive memory trials (Fig. 6a,b, left and middle).

In addition, we found a significant fraction of cells with increased but not decreased theta-band phase coupling during contraversive as compared to ipsiversive trials (ACC units: 34/353=9.6%, *P*<0.001; FEF units: 15/209=7.2%, *P*<0.001, Binomial test; Fig. 5a,b, right). Furthermore, we observed that the average spike-LFP phase locking was increased during contraversive as compared to ipsiversive trials in a subset of theta frequencies (3–4 Hz; Fig. 6a,b, right).

**Figure 7:**
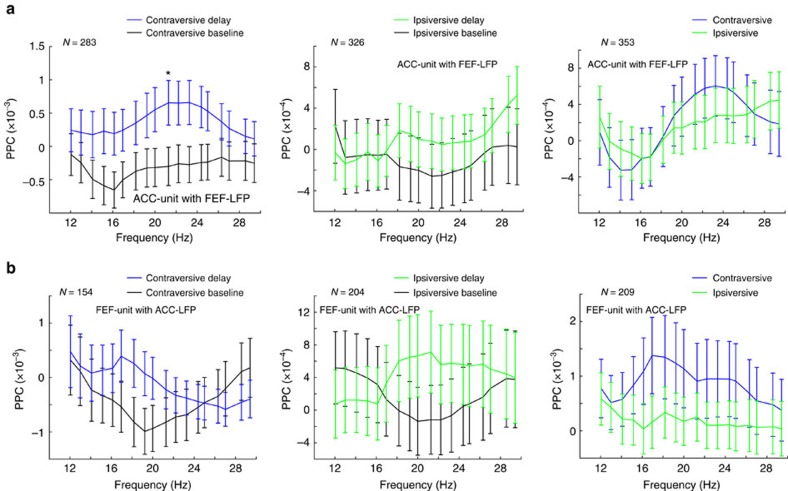
Pairwise phase consistencies (PPCs) across beta band. (**a**) PPC spike-field coherence spectrum of the population of the ACC-unit with FEF-LFP pairs across the beta frequency range. Comparison between baseline and delay of contraversive saccades (left), comparison between baseline and delay of ipsiversive saccades (middle), and comparison between the contra- and ipsi-versive saccades in the delay period (right). (**b**) PPC spike-field coherence spectrum of the population of the FEF-unit with ACC-LFP pairs across the beta frequency range. Comparison between baseline and delay of contraversive saccades (left), comparison between baseline and delay of ipsiversive saccades (middle), and comparison between the contra- and ipsiversive saccades in the delay period (right). Error bars denote s.e.m. in all panels. *P<0.05, paired *t*-test.

We also found that a small but significant fraction of ACC-unit with FEF-LFP pairs and FEF-unit with ACC-LFP pairs showed increased phase coupling in the beta band (12–30 Hz) in the delay period versus baseline of both contraversive (ACC units: 17/283=6.0%, *P*<0.001; FEF units: 15/154=9.8%, *P*<0.001, Binomial test) and ipsiversive trials (ACC units: 22/326=6.8%, *P*<0.001; FEF units: 15/204=7.4%, *P*<0.001, Binomial test), whereas we found no evidence for significant decreases (Fig. 5a,b, left and middle). Although we found a trend for increased average spike-field phase locking at beta frequencies during the delay as compared to the baseline period, this effect only reached significance for the population of ACC-unit with FEF-LFP pairs (Fig. 7a, left). Furthermore, in contrast to the theta band, we found no evidence for significant changes in beta-band spike-LFP phase locking between ipsi- and contraversive trials for ACC-unit with FEF-LFP (Fig. 5a, right). A small but significant fraction of FEF cells exhibited increased beta-band phase locking to ACC LFPs in ipsiversive as compared to contraversive trials (17/209=8.1%, *P*<0.001, Binomial test) (Fig. 5b right). Moreover, we did not observe a significant difference in the average spike-LFP phase locking during the delay periods of contraversive as compared to ipsiversive trials across beta-band frequencies (Fig. 7a,b, right). Finally, we tested the spike-field coupling of the ACC and FEF units with FEF and ACC LFP, respectively, across delta, alpha, and gamma bands and we did not observe significant effects (Fig. 6 and Supplementary Figs 5-7).

We also tested for possible influences of firing rate differences between units on the observed spike LFP synchronization. We repeated the spike-field analysis, but only including in the analysis those units with no significant difference of spike rates between the tested conditions. The obtained effects were still statistically significant (see ref. 25 for a detailed description).

In summary, we found evidence for positive modulations in theta and beta phase coupling both during contra- and ipsiversive trials, consistent with the LFP-LFP phase-synchronization analysis. We found that increases in theta-band spike-LFP phase coupling were more prominent during contraversive than ipsiversive memory trials.

## Discussion

Here we have reported that neural circuits in the ACC and the FEF synchronize the phases of theta- and beta-specific activity during the short-term retention of stimulus locations in a working memory task. This working memory-induced interareal synchronization (1) was evident in more than half of the LFP-LFP recording pairs, (2) translated to spiking activity in the ACC and FEF with significant interareal spike-LFP synchronization in both anatomical directions, that is, with spikes from ACC coupled to LFPs in FEF and with FEF spikes coupling to LFPs from ACC, (3) indexed correct versus erroneous working memory performance, and (4) exhibited asymmetric directionality, with stronger influence of ACC over FEF than vice versa. These findings provide evidence that functional interactions between brain areas implementing (associated with FEF) and biasing (associated with ACC) working memory and also higher-order cognitive performance proceed through phase-synchronized activation at band-limited theta and beta rhythmic activity^15^,^16^,^26^. These results indicate how larger working memory networks coordinate their activity and constrain the possible cell and circuit mechanisms that underlie successful working memory performance^7^.

Neuronal circuits in FEF are well known to encode target locations for overt orienting and covert stimulus selection during working memory, sustained attention, and visual search tasks (reviewed, for example, in ref. 18). During working memory and stimulus selection, the long-range synchronization of FEF with intraparietal areas takes place at characteristic beta-range frequencies^17^,^27^,^28^, and at higher gamma-band frequencies with visual area V4 neurons that share similar spatial tuning to target locations^29^,^30^. Beyond these beta- and gamma-band-mediated interactions across frontoposterior brain areas with spatially tuned neuronal circuits, it has been unknown how more anterior structures including circuits in the ACC are linked to the FEF during ongoing frontoparietal network activation. Our findings answer this question by documenting theta (3–9 Hz) and beta (12–30 Hz) frequency-specific synchronization between neural circuits in FEF and in ACC. In this regard, WPLI-debiased analysis and Granger-causality analysis demonstrated a concurrent theta- and beta-band increase in the delay period compared to the baseline across the majority of channel pairs. Band-limited synchronization at theta and beta frequencies could provide a temporal reference for coordinating spiking activity between both areas^17^,^31^. We discuss the results on beta- and theta-band synchronization separately in what follows.

Transiently emerging LFP activity at a 3–9 Hz theta frequency band has been shown to characterize ACC circuits during preparatory states^32^,^33^ and during top-down controlled stimulus selection^16^. Complementary to the latter study findings, theta rhythmic activation in our task emerged in response to a sample stimulus that served as a cue to select a spatial target location as later saccadic target location. The theta rhythmic synchronization of ACC circuits with the FEF that we observed is thus directly related to the cognitive demands to establish and maintain an internal spatial target representation. Consistent with this functional interpretation we found that a failure to maintain the target location in working memory was evident in reduced theta rhythmic interactions of ACC and FEF, suggesting that the phase synchronization between ACC and FEF is functionally important to successfully achieve the behavioural goal. Notably, similar to a previous study on theta activation in the ACC^16^, locally confined LFP theta power modulations were not as informative to predict successful top-down performance compared to interareal theta-band interactions (Fig. 4; see also ref. 16). We believe that this set of results reveal that the mere activation of theta rhythmic circuit motifs within the ACC and FEF are not sufficient to support adaptive behaviour^14^. Rather, these findings highlight that theta rhythmic circuit motifs need to be coordinated between areas to successfully maintain an internally generated top-down network state^7^,^26^,^34^.

Could the observed theta-band synchronization between ACC and FEF be part of a larger functional network? We observed enhanced ACC-FEF phase synchronization during a working memory task that was originally used to discover persistent working memory activity in lateral prefrontal cortex (area 46/9 and anterior area 8b)^35^. Several studies have shown that this lateral PFC hot spot of working memory does not act as a discrete working memory module, but that it acts within a broad working memory network of brain areas that includes the FEF and the ACC^36^. Such a network perspective is consistent with the dense anatomical interconnectivity of ACC, FEF, and lateral PFC^37^. Functionally, this network perspective of working memory is supported by a study that documented increased 3–9 Hz theta LFP power and increased PFC-to-V4 theta-band phase synchronization during the working memory delay of a match-to-sample task^15^. The convergence of these major findings about the interareal signature underlying successful working memory performance opens the possibility that all four brain areas, lateral PFC, FEF, ACC, and V4, phase synchronize their local activities to a common theta rhythm during successful maintenance of working memory that would be measurable if all areas were recorded simultaneously. An important consequence of this scenario is that cellular and circuit mechanisms that generate and sustain theta rhythmic circuit activation would be key mechanisms underlying the interareal coordination of working memory representations^14^,^38^.

We found that a moderate fraction of ACC and FEF units synchronized to theta LFP activity at distant sites (FEF and ACC, respectively) more strongly during the working memory delay than during the predelay baseline period (see Figs 5 and 6). This increase was particularly prominent for contraversive memory locations, indicating that interareal spike-LFP synchronization carried top-down information similar to previous reports^15^,^32^. These observations suggest that theta phases measured in LFP activity in the ACC and in the FEF during the delay period can be conceived of as a direct measure of coordination of spiking activity in the local circuits^16^. Theta phase indexed the spike-LFP synchronization from ACC to FEF and from FEF to ACC during retention of target locations. We speculate that this reciprocal interaction indexes the exchange of area-specific information. For example, FEF spikes carry location-specific information about contralateral targets that could be conveyed to ACC circuits. ACC neurons typically containing spatially tuned targets only when those targets are linked to outcomes for top-down controlled behaviour such as reward or attentional control demands^39^,^40^. A linkage to these representations in the ACC could be crucial to prevent premature responding during the working memory delay consistent with previous human and monkey results, suggesting that ACC activation prevents impulsive responding^41^,^42^. Indeed, it has been previously shown that neuronal spiking output can modulate the oscillatory activity by influencing the postsynaptic potentials^43^. Thus, spiking activity that is synchronized between ACC and FEF could index the ongoing coupling of task relevant information required for successful working memory performance.

The role of frontal midline theta in working memory has been previously demonstrated in human studies and the ACC has been suggested as its main source^44^. Moreover, the ACC has long been implicated in performance or conflict monitoring^45^. In this regard, human studies have suggested that frontal midline theta is involved in the processing of conflict and errors^46^. These reports suggest that the theta band plays a major role in the cognitive functioning of the ACC in humans, macaques, and rodents and our results provide further support for this claim.

As mentioned in the introduction, beta-band synchronization is suggested to be involved in long-range transmission of information between brain areas^17^,^18^,^19^,^20^. In addition to theta-band modulations, our study found a similarly prominent modulation of delay period activity in the beta frequency band. Enhanced ACC-FEF beta-band-specific coherence (1) was almost as prevalent as theta coherence (beta: 49% versus theta: 66% of pairs), (2) showed significantly enhanced Granger causality that pointed to a stronger ACC influence over FEF than vice versa, and (3) was significantly reduced on error trials. These signatures resonate well with beta-band spike-field coherence between FEF and parietal cortex and between ACC with lateral prefrontal cortex during goal-directed task performance^14^,^17^. Moreover, a previous study has documented that beta-band-specific activation is involved in the directional influence of the higher-order association cortices over primary motor areas^21^. Our study suggests that the same frequency that characterizes these frontoparietal interactions also incorporates interactions with medial frontal (ACC) and oculomotor (FEF) circuits. In a recent study, it has been shown that beta-band synchrony is involved in cognitive and motor control during gait adaptation^47^. This report is in line with our findings suggesting the involvement of the ACC-FEF beta-band synchronization in sensorimotor mapping. Furthermore, interareal beta synchrony is suggested to be enhanced with selective attention^20^,^27^,^28^. We observed increased beta synchrony both in the delay period of the memory-guided saccade task and the preparatory period of the anti-saccade task. These findings suggest that the increased cognitive and attention demands of the tasks could underlie increased beta-band synchronization between these areas. It will be an important task for future studies to characterize the extent and distribution of such a putative large-scale beta frequency band network and to disentangle the information carried in frequency-specific coupling in such a beta network when compared to the network of brain areas that synchronize at theta frequency band^7^.

One possible mechanism for ACC-FEF phase synchronization could be based on interactions of a subclass of parvalbumin expressing, fast spiking interneurons, and subsets of pyramidal cells showing resonance to theta rhythmic inhibition^48^. Stark and co-workers^37^ have documented that these interneurons induce theta rhythmic firing in pyramidal cells in the medial prefrontal cortex of rodents that is considered to be partly functional analogous to primate anterior cingulate cortices. Importantly, during 3–10 Hz theta rhythmic inhibition, pyramidal cell firing was not reduced as would be expected for an inhibitory regime, and even showed increased postinhibitory firing, essentially implementing a theta-mediated amplification of firing^48^. The amplification of spike output may facilitate and sustain long-range theta coherence between anterior cingulate, hippocampal, and parietal and medial prefrontal structures of the rodent^49^. Theta synchronization emerges in rodents during choice tasks that require working memory recall of spatial reward associations similar to spatial target associations that need to be maintained in our oculomotor response task^50^. We believe that these rodent studies could therefore be informative about the cellular basis of working memory networks in primates. Consistent with this suggestion, a recent study in macaque ACC and lateral PFC identified two functional subclasses of a total of seven separable classes, one putative interneuron subclass and one putative pyramidal cell subclass, that showed a particular prominent phase synchronization with LFP's at theta-band frequencies^51^. These theta-band neurons segregated from other functional classes of neurons that either did not synchronize to the LFP at any frequency or that preferred to phase align their spiking activity with beta-band oscillatory activity instead of theta frequencies^51^. Taken together, these findings suggest that frequency-specific synchronization of firing could be established by subsets of interneurons and pyramidal cells that share intrinsic resonance properties, and that are largely segregated from neuron populations that synchronize their firing to LFP activity at, for example, beta-band frequencies^14^,^52^.

We observed that the theta-band Granger causality increased in the delay period in both ACC→FEF and also FEF→ACC directions. This could indicate that theta-band synchrony is involved in both top-down and bottom-up processes, as has been described previously^15^,^53^. On the other hand, the beta Granger causality only increased in the ACC→FEF direction. This is in line with previous studies indicating a role of beta band in top-down control, for a review see ref. 54. These interpretations correspond with our findings from field-field coherence and spike-field coherence analysis.

**Figure 8:**
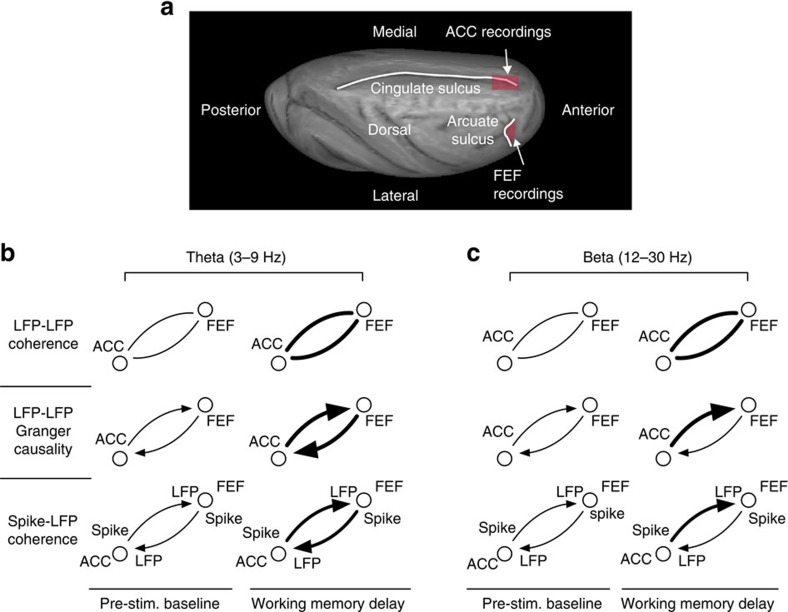
Illustration of recorded brain area locations and summary of main interareal ACC-FEF modulations observed in this study. (**a**) ACC and FEF recording locations (in red shading) shown on a rendering of a semi-inflated macaque brain. (**b**,**c**) Illustration of main interareal effects in the theta (**b**) and beta (**c**) band with the thickness of connections indicating the strength or prevalence of the effects. LFP-LFP coherence (top row) was modulated during the delay in >75% of LFP-LFP pairs in both frequencies (with increased coherence in the largest majority). Granger causality (middle row) increased during the delay for both ACC to FEF, and FEF to ACC directions (more pronounced in theta band), but the ACC to FEF Granger-causal flow was stronger than FEF to ACC Granger-causal flow at both theta and beta frequencies. Spike-LFP coherence (bottom row) increased for both directions during the delay in the theta band, but was different between delay and baseline merely in one beta frequency bin (at 22 Hz) for ACC spike to FEF LFP sites for contraversive saccades. Reduced interareal modulation prior to error commission was evident in both frequencies across different measures and is described in the text.

In summary, the increased ACC-FEF phase synchrony and also increased influence of the ACC over FEF during the delay period suggests that the ACC-FEF interaction serves a functional role. The increased ACC unit-FEF LFP coupling in the delay period of the contraversive saccades could indicate that the ACC biases FEF to generate a contraversive saccade (Fig. 8). Our results also suggest that FEF sends target location (or saccade target) signals to the ACC through increased FEF unit-ACC LFP coupling in the delay period of contraversive saccades (Fig. 8). We believe that selective theta and beta frequency coherence of the FEF and ACC during working memory delays reveals how top-down information is actively coordinated to ensure the optimal harvesting of rewards during top-down controlled behaviour. We speculate that theta cycles may provide the critical reference for neuronal spikes in distributed brain systems to code for goal- and choice-relevant information^14^,^31^,^55^.

## Methods

### Subjects

Two male adult macaque monkeys (*Macaca mulatta* and *Macaca fascicularis*) weighing 7–9 kg were subjects in this study. Recording chambers were implanted over the right arcuate sulcus and right anterior cingulate sulcus based on previously obtained MRIs. Details for the surgical procedures have been described previously^56^. Postsurgical MRIs were obtained to confirm the location of the recording chambers and to allow reconstruction of the recording sites. All experimental procedures and animal care were implemented in accordance with the guidelines of the Canadian Council of Animal Care policy on the care and use of laboratory animals and an ethics protocol approved by the Animal Users Subcommittee of the University of Western Ontario Council on Animal Care. The monkeys were under close supervision by the university veterinarians.

### Behavioural task

Animals were trained to perform a standard memory-guided saccade (Fig. 1a). Each trial started with the presentation of a white central fixation point (0.15°). Once monkeys fixated this spot within a 0.5° × 0.5° window for 500 ms, a white target stimulus (0.15°) was presented for 100 ms with equal probability in one of eight cardinal directions (0°, 45°, 90°, 135°, 180°, 225°, 270°, and 315°) at a distance of 9° from the central fixation point. The animals were required to maintain fixation during the stimulus presentation and during the 1000ms period following stimulus presentation, which we define as the delay period of the task. During the delay period, the central fixation point remained illuminated and the target stimulus was not visible. The offset of the central fixation point instructed the monkeys to perform a saccade toward the remembered target location. To obtain a water reward, the monkeys were required to perform a saccade within 500 ms after the offset of the central fixation point toward the remembered location (window of 5° × 5°) and to maintain fixation at the remembered target location for a random period of 300–600 ms. If the animal correctly performed all the required steps, the target stimulus reappeared and the animal received a water reward (Fig. 1a). Eye movements were recorded at 500 and 1,000 Hz using video eye trackers (EyeLink II and EyeLink 1,000, Kanata, ON, Canada). Presentation of the behavioural stimuli, monitoring of the responses, and reward delivery were controlled using CORTEX as the experimental control software (NIMH, Bethesda, MA, USA). Task events, vertical and horizontal eye positions, and digitized neural signals were stored together using either a Plexon MAP or Omniplex System (Plexon Inc., Dallas, TX). We visually inspected the eye traces and the trials in which the eye movement trajectory deviated from the normal pathway were excluded. Monkey 1 correctly performed 94.68% and monkey 2 correctly performed 89.97% of memory-guided saccade trials.

The animals did not make many errors in the memory-guided saccade task and this makes the analysis of performance-related effects challenging. To overcome this shortcoming of the memory-guided saccade task, we also used a randomly interleaved pro-/anti-saccade task and the anti-saccade task led to more direction error trials. In this task, each trial started with the appearance of a central fixation point (0.15°) the colour of which instructed the monkeys to perform a pro- or anti-saccade on stimulus presentation. The fixation point remained illuminated for a period of 1,100–1,400 ms, which we designate as the preparatory period. The monkeys were required to maintain fixation during the preparatory period and then the target stimulus appeared at a distance of 9° either to the right or left of the central fixation point. In pro-saccade trials, the monkeys were required to saccade towards the target stimulus, and in anti-saccade trials, they had to make a saccade towards the mirror location of the target stimulus (Fig. 1a). To obtain a water reward, the monkeys were required to maintain fixation at the target location for a random period of 300–600 ms. Monkey 1 correctly performed 76.85% and monkey 2 correctly performed 82.63% of anti-saccade trials.

### Electrophysiological recordings

We simultaneously recorded the LFP and unit activity in the ACC and FEF while monkeys performed a memory-guided saccade task and a randomly interleaved pro-/anti-saccade task (Fig. 1a–c). FEF and ACC recordings were performed by advancing tungsten microelectrodes (FHC, Bowdoinham, ME) through guide tubes into the brain using software-controlled precision microdrives (NAN Instruments Ltd, Israel) on a daily basis. The microdrives were assembled on a plastic grid with 1 mm interhole distance. At the beginning of each experimental session, 2–4 electrodes were advanced into the FEF and ACC. The coordinates of the ACC recording sites for monkey 1 were 5–17 mm anterior, 8–12 mm dorsal, and 2–4 mm to the right side of the anterior commissure. Similarly, the coordinates of the ACC recording sites for monkey 2 were 7–17 mm anterior, 12–16 mm dorsal, and 2–4 mm to the right side of the anterior commissure. The coordinates of the FEF recording sites for monkey 1 were 1 mm anterior, 8–12 mm dorsal, and 13–16 mm to the right side of the anterior commissure. The coordinates of the FEF recording sites for monkey 2 were 2–4 mm anterior, 10–16 mm dorsal, and 12–16 mm to the right side of the anterior commissure. The ACC recording sites included areas 24d, 24b, and 10m according to the composite monkey brain atlas of Van Essen *et al*.^57^. The proper location of the FEF electrodes was confirmed by electrical microstimulation as described previously^2^ (train duration of 70 ms set at 300 Hz, biphasic pulses of 0.2 ms duration, current <50 μA). Supplementary Figure 1 shows a reconstruction of the ACC and FEF recording sites.

The signal from each electrode was preamplified (Plexon Inc.) and band-pass filtered between 0.5–250 Hz for LFP signals and between 250 and 8,000 Hz for the single /multiunits and digitized at the frequency of 40 kHz. Each area's signal was referenced separately against the corresponding recording chamber. The sampling rate for LFP signals was set at 1,000 Hz. ACC and FEF multineuron activity was isolated offline by applying a threshold±3–5 s.d. above or below the average raw signal to separate the multineuron activity from noise. We further visually inspected the waveforms to confirm that this procedure resulted in normal waveforms and the multiunits with abnormal waveform shape were excluded from the analysis. We will refer to multineuron activity as units throughout the paper.

### Spectral power analysis

All of the data analyses were performed with custom-written Matlab code (Mathworks, Natick, MA) using the fieldtrip toolbox ( http://www.ru.nl/fcdonders/fieldtrip/)^58^. We first performed an artefact rejection procedure. In this procedure, the trials in which the LFP power exceeded 4 s.d. from the average were excluded from the analysis. Then, we performed fast Fourier transformation following hanning tapering of the data^59^ (a multitaper method with a discrete prolate spheroidal sequence was used to analyse 40–100 Hz frequencies) around the time of the target stimulus onset and the delay period. The analysis window started at 1,500 ms prior to and ended at 1200, ms after the target stimulus onset. The analysis time window did not have any overlap with the reward presentation to avoid contamination of the data with possible reward consumption artefacts. We performed the frequency analysis using a 670 ms sliding time window in 50 ms steps. The power spectra for individual channels were *z*-score normalized using a baseline of 500–1,000 ms prior to the fixation point onset in both memory-guided saccade task and pro-/anti-saccade task.

### Field-field coherence analysis

We performed a phase coherence analysis between FEF and ACC electrodes using WPLI-debiased^34^,^60^,^61^ (see ref. 61 for a detailed mathematical definition of the method). Briefly, WPLI is exclusively based on the imaginary component of the cross-spectrum and it is insensitive to volume conduction from a single source or a common reference. Furthermore, the WPLI is more robust to noise and is invariant when two dependent sources mix linearly and thus it is more sensitive in detection of the true interactions between two signals^60^,^61^. WPLI is defined as:


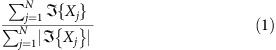


Here 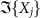 is the imaginary component of the cross-spectrum in the *j*th trial. Sample size is a concern when using WPLI and hence we used WPLI-debiased, which estimates the squared WPLI. WPLI-debiased has negligible sample size bias for data sets of small sample size^61^. To test for the difference in WPLI-debiased coherence across experimental conditions, we first averaged the WPLI-debiased theta-band coherence across the appropriate time window for each channel pair (for example, 400–1,100 ms following the target stimulus as the delay related WPLI-debiased theta coherence and 700 ms prior to fixation point onset as the baseline WPLI-debiased coherence in the memory-guided saccade task). Then, we performed a *t*-test to investigate whether there is a statistically significant difference across experimental conditions. We performed the comparison of WPLI-debiased coherence between correct and error trials with the adjusted number of correct trials relative to errors and the results showed significant effects as we have reported in this manuscript. We also used a permutation test to further validate the results obtained by the *t*-tests. In this test, we pooled the data from two conditions and randomly split the pooled data into two groups with the same sample sizes for a minimum of 1,000 times. Afterwards, we obtained a distribution of the difference in the means of the shuffled groups. If the difference of the means of the original samples fell at least >95% of the generated distribution (>97.5% for two-tailed tests), we considered the difference between groups as significant. We obtained statistically significant results in all the comparisons that *t*-test yielded statistical significance; thus, we could validate the *t*-test results with this permutation test.

The WPLI-debiased method outputs a single value for a given trial set and thus, we cannot perform a traditional statistics to test whether there is a difference in WPLI-debiased coherence between the baseline and delay period of a single channel pair. To test for a statistically significant difference in coherence between baseline (700 ms before fixation onset) and delay (400–1,100 ms after stimulus onset) period in a single channel pair, we used a bootstrapping approach. We created 1,000 bootstraps with replacement of the original trial set and obtained a distribution of the WPLI-debiased for the baseline and delay periods. The difference between baseline and delay was determined to be significant if the mean of the WPLI-debiased in the delay period fell above or below the 99.9th percentile of the WPLI-debiased distribution of the baseline period. To further confirm the results of the bootstrapping procedure, we pooled the baseline and delay WPLI-debiased results and randomly assigned them into two groups. Then, we tested whether the mean difference between the original baseline and delay period falls above or below the 99.9th percentile of the mean difference of the randomly generated groups. The results of this procedure yielded nearly identical results to the bootstrapping procedure we have described above.

We have subtracted the evoked response from the raw data to investigate whether or not the observed phase synchronization is due to the induced oscillations. To further examine whether the observed synchronization was due to the induced oscillations, we have subtracted the evoked response using a method described by Truccolo *et al*.^23^ We have included these results in Supplementary Fig. 3.

### Granger-causality analysis

To analyse the directionality of interactions, we performed Granger-causality analysis. Granger-causality analysis is based on modelling the two time series as a vector autoregressive model,


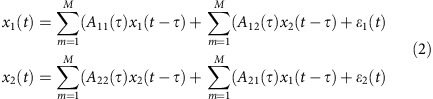


In this equation, the two signals are predicted by a linear combination of their own past values, and the past values of the other time series. The residual errors (unexplained variance) are captured by the variables 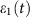 and 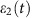, with covariance matrix 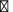. We estimated the coefficients for the VAR model by setting the order *M*=32 and using the *armorf* function in the BSMART toolbox^62^ to find the least-squares solution to equation (2). We then used the standard decomposition of Granger's time domain causality into frequency-domain Granger causality, as developed by Geweke *et al*.^63^. Fourier transformation of equation (2) yields the spectral decomposition


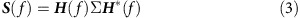


The frequency-domain Granger causality from *x*_2_ to *x*_1_ is now defined as


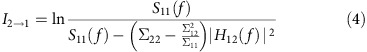


This expression can be understood as the log fraction of intrinsic power in the first signal at frequency *f* over the amount of power that remains after the predictions from the second signal have been factored in.

It is well established that Granger causality can be sensitive to both uncorrelated and correlated noise that is superimposed on the measurements of the signals of interest. This can lead to a substantial amount of false alarms in the identification of Granger-causality relationships (see refs 64, 65, 66, 67, 68). An effective control for the noise problem can be achieved by using the time reversal of signals^67^. If the Granger causality from *x*_1_ to *x*_2_ is stronger than the Granger causality from *x*_2_ to *x*_1_, then we expect that the reverse holds true when we time reverse the signals^67^. Thus, we only accept conclusions about the asymmetry of Granger causality when both conditions hold true^67^. For the analysis of the present dataset, we determined for each channel combination whether the asymmetry in Granger-causality values in the theta-frequency range flipped after time reversing the signals. We only selected those ACC-FEF channel combinations for which this held true. In other words, if an ACC (FEF) channel tended to Granger cause the FEF (ACC) channel in the theta range, then we expected that the FEF (ACC) channel would Granger cause the ACC (FEF) channel after time reversing the signals. As shown by Vinck *et al*.^67^, this provides a highly effective procedure to diminish the influence of correlated and uncorrelated noise.

The Granger-causal influence of the ACC over FEF is shown as GACC→FEF and the Granger-causal influence of FEF over ACC is denoted as GFEF→ACC. The overall influence of ACC over FEF was derived by implementing the (GACC→FEF−GFEF→ACC) equation. The results shown in Fig. 3f,h are obtained by implementing the (GACC→FEF−GFEF→ACC) equation. The results shown in Supplementary Fig. 4A depicts GACC→FEF, Supplementary Fig. 4B shows GFEF→ACC, and Supplementary Fig. 4C shows both GACC→FEF (blue) and GFEF→ACC (red). We tested the difference between the baseline (700 ms before fixation onset) and the delay period (400–1,100 ms following stimulus onset), using a *t*-test. We further confirmed the results of the *t*-test by implementing a permutation test. The permutation test procedure is described in detail above.

We also shuffled the time-frequency transformed raw data and computed the corresponding Granger values to obtain the chance level Granger. Then we compared the chance level GACC→FEF and GFEF→ACC values to the original Granger values from the non-shuffled data set. We observed that across theta and beta frequencies, both the GACC→FEF and GFEF→ACC values in the baseline and delay periods were above the chance level.

### Spike-field coherence analysis

We used the PPC as a measure of spike-field synchrony; for a detailed mathematical definition see ref. 24. Briefly, the PPC measure is robust to variations in the neuronal spike rate and to possible interdependencies of the spike-LFP phases^25^. It is defined as


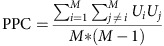


Here *M* is the number of spikes, *U*_*i*_ is the vector describing the *x* and *y* component of the spike-LFP phase for the *i*th. We only included units with at least 50 spiking events in the tested conditions to obtain a reliable and robust results and increase the statistical power of the analysis as has been shown before^15^,^69^. We performed a paired *t*-test and also a permutation test to probe the significant difference of the PPC across experimental conditions. We have described the permutation procedure above. To correct for multiple comparisons, we have implemented the false discovery rate algorithm as described by Benjamini and Yekutieli^70^.

To test for significant spike-field coupling across single ACC-unit with FEF-LFP and FEF-unit with ACC-LFP, we performed a permutation test. We first randomly shuffled the spikes across the tested conditions (for example, baseline and delay) and obtained a distribution of the PPC values in the randomized experimental conditions. Then, we tested whether the PPC values of the original non-randomized conditions fell above or below the 2.5th percentile of the randomization distribution. If so, we designated that unit-LFP pair as having a significant modulation across the tested conditions.

### Data availability

We have not made the raw data available. However, the computer codes used to perform data analysis could be provided on request.

## Additional information

**How to cite this article:** Babapoor-Farrokhran, S. *et al*. Theta and beta synchrony coordinate frontal eye fields and anterior cingulate cortex during sensorimotor mapping. *Nat. Commun.* **8,** 13967 doi: 10.1038/ncomms13967 (2017).

## Supplementary Material

Supplementary Information Supplementary Figures and Supplementary References
