# Supplementary material for: Theta and beta synchrony coordinate frontal eye fields and anterior cingulate cortex during sensorimotor mapping
Source: Nat Commun. 2017 Feb 7;8:13967. doi: 10.1038/ncomms13967 (PMC5309702; doi:10.1038/ncomms13967)
Supplement: Supplementary Information — Supplementary Figures and Supplementary References [file ncomms13967-s1.pdf]

Supplementary Figures:

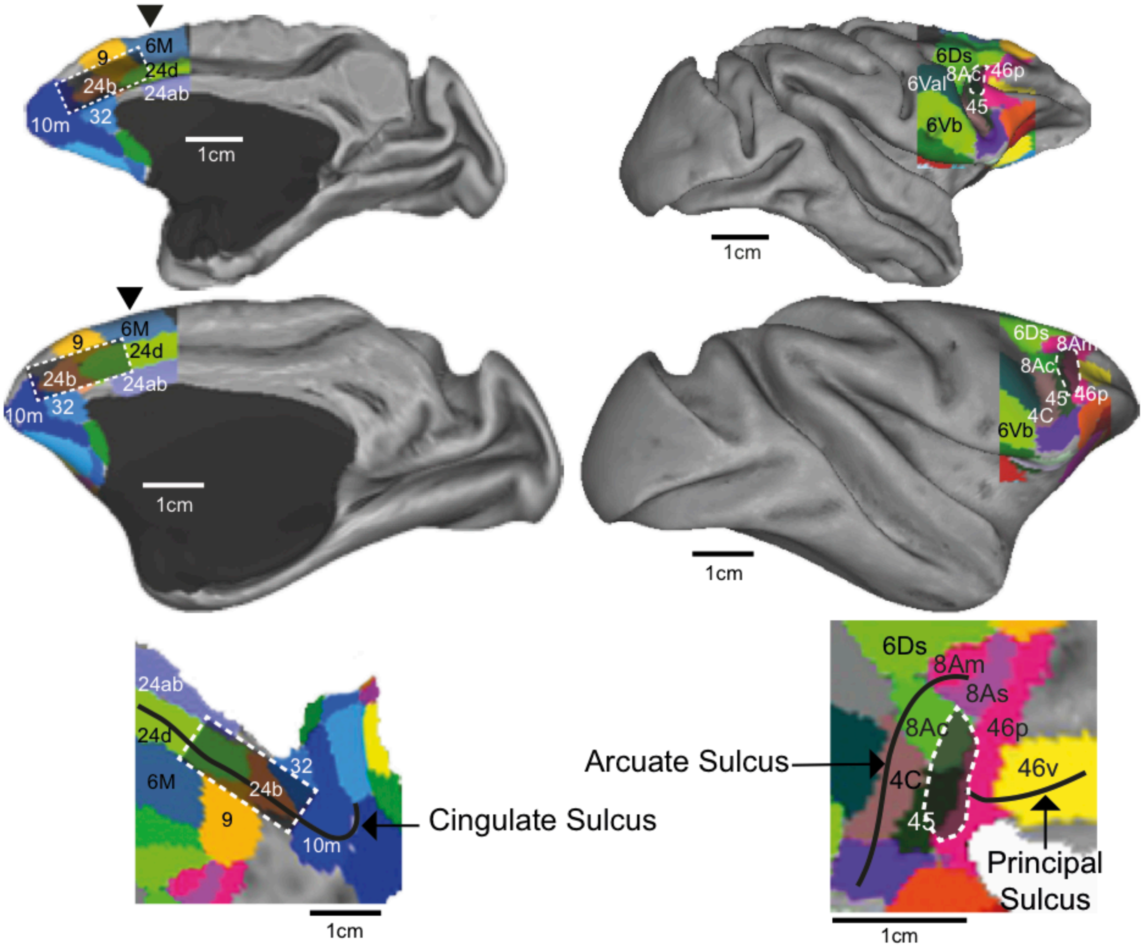

Supplementary Figure 1. Schematic reconstruction of the recording sites. The recording sites are overlaid on the F99 surface (using Caret software, Van Essen lab) according to the composite atlas of Van Essen et al. (2012)[1]. The left column is the reconstruction of the ACC recording sites shown on a fiducial (up), inflated (middle) and flat surface map. The arrows on the top and middle panels denote the location of the angle of the Arcuate sulcus. Similarly, the left column sows the reconstruction of the FEF recording sites.

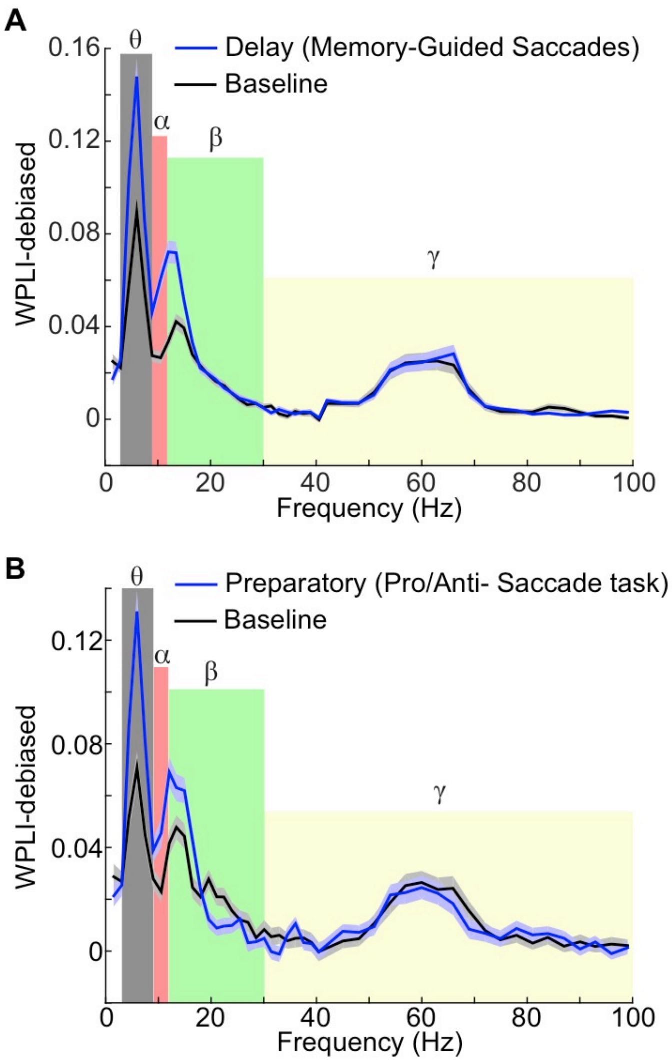

Supplementary Figure 2. WPLI-debiased spectrum. (A) The phase synchronization spectrum between ACC and FEF in memory-guided saccades is depicted across all recording pairs (n=674). The panel shows larger ACC-FEF phase synchronization across theta and beta bands in the delay period (400-1100 ms following the stimulus onset, blue) compared to the baseline (700 ms prior to the fixation onset, black). (B) The phase synchronization spectrum between ACC and FEF in pro-/anti-saccades is depicted across all recording pairs (n=674). The panel shows larger ACC-FEF phase synchronization across theta and beta bands in the preparatory period (400-1100 ms following the fixation onset, blue) compared to the baseline (700 ms prior to the fixation onset, black). The shading shows +/- SEM.

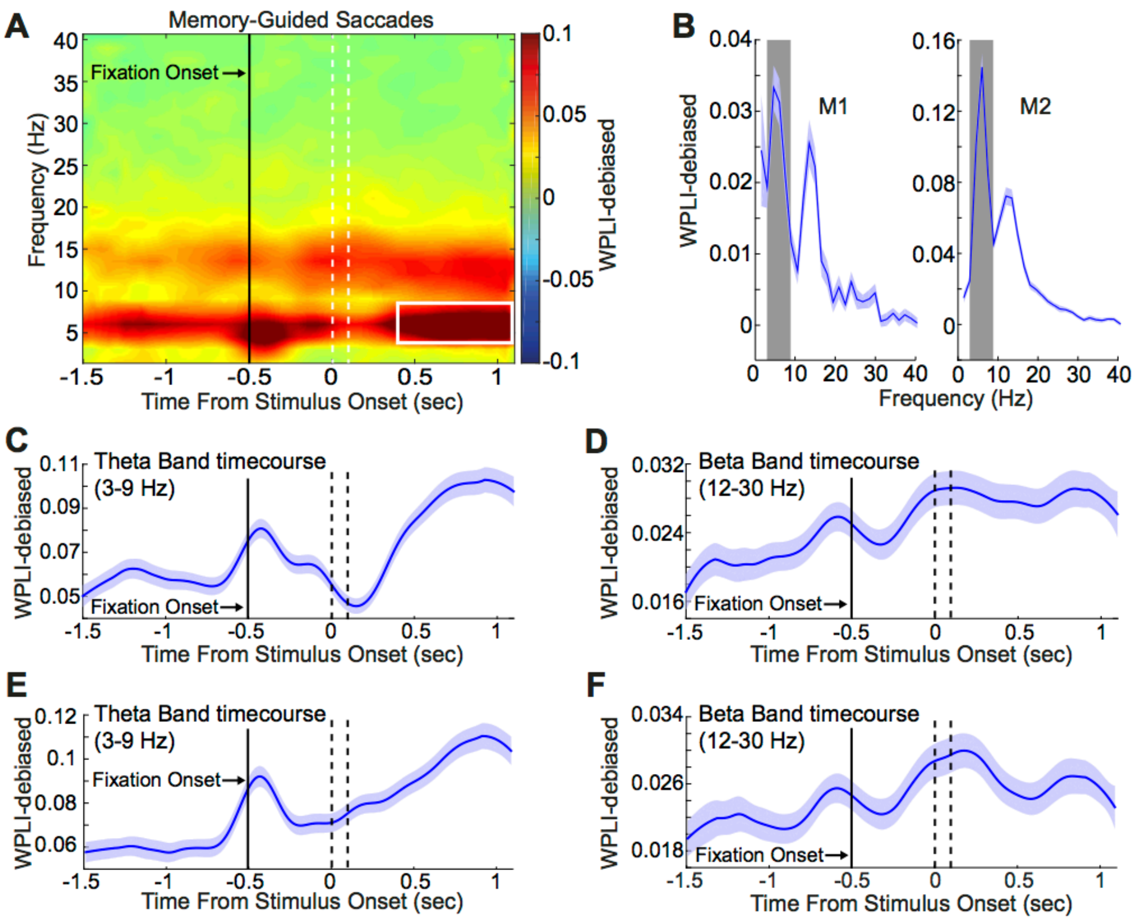

Supplementary Figure 3. Increased theta- and beta- coherence between ACC and FEF after event-related signal subtraction. (A) Time-Frequency spectra of the wpli-debiased coherence between the FEF and ACC in memory-guided saccade task after subtraction of the averaged event-related potentials from the raw signal. The white contour shows the area in which the subsequent analyses were performed (see Methods). The dashed lines demarcate the time of the onset and offset of the target stimulus. (B) WPLI-debiased FEF-ACC coherence spectrum of the individual monkeys in the delay period across all recording pairs (n=674) after the subtraction of evoked response. (C) Theta band (3-9 Hz) time course of the ACC/FEF WPLI-debiased phase synchronization after subtraction of evoked response. (D) Beta band (12-30 Hz) time course of the ACC/FEF WPLI-debiased phase synchronization after subtraction of evoked response. (E) Theta band (3-9 Hz) time course of the ACC/FEF WPLI-debiased phase synchronization after subtraction of evoked response using a method described by Truccolo et al.[2] (F) Beta band (12-30 Hz) time course of the ACC/FEF WPLI-debiased phase synchronization after subtraction of evoked response using a method described by Truccolo et al.[2].

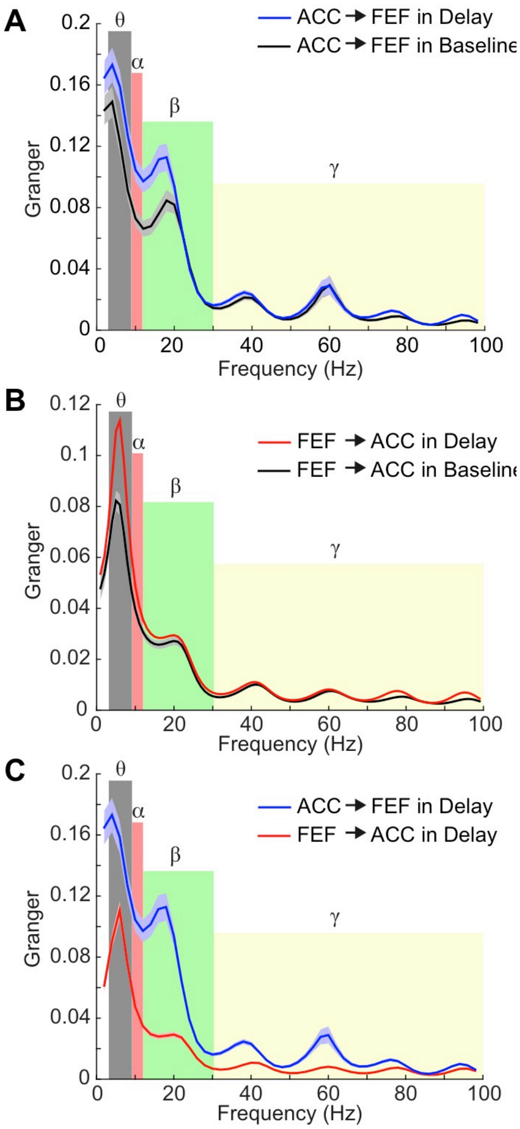

Supplementary Figure 4. Granger causality spectrum between ACC and FEF. (A) Influence of ACC over FEF in the delay period (400-1100 ms following the stimulus onset, blue) and the baseline (700 ms prior to fixation onset, black). The influence of ACC over FEF is larger in the delay period across theta and beta frequency range. (B) Influence of FEF over ACC in the delay period (red) and the baseline (black). The influence of FEF over ACC is larger in the delay period across theta and beta frequency range. (C) Influence of ACC over FEF (blue) is higher than the influence of FEF over ACC (red) in the delay period across theta and beta frequency range. The shading depicts +/- SEM. The ACC-FEF channel pairs (n=275) that displayed reversed Granger causality following the reversal of time series are included in this figure.

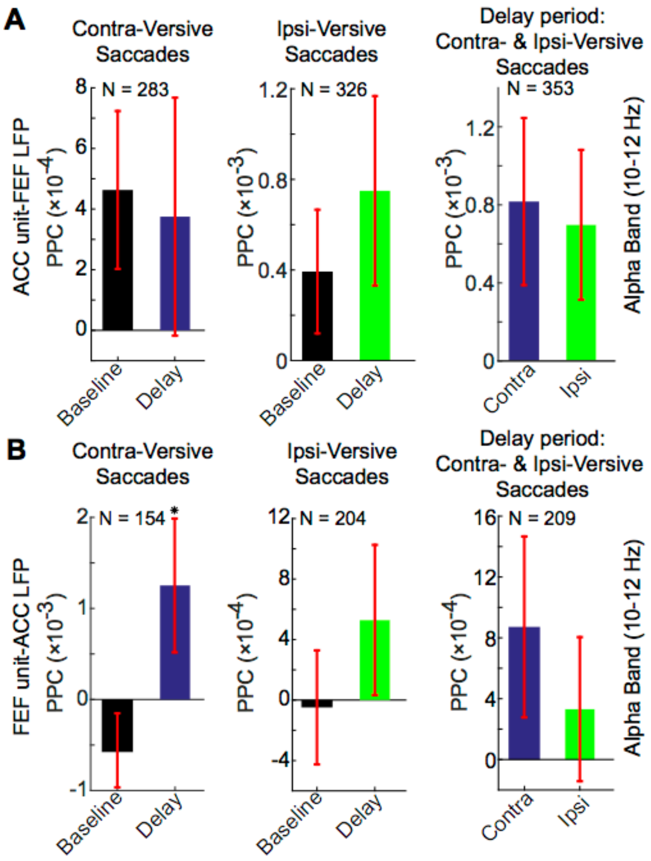

Supplementary Figure 5. Pairwise phase consistencies (PPCs) across alpha band. (A) PPC spike-field coherence spectrum of the population of the ACC units with the LFP's recorded in the FEF across the alpha frequency range. Comparison between baseline and delay of contra-versive saccades (left), comparison between baseline and delay of ipsi-versive saccades (middle) and comparison between the contra- and ipsi-versive saccades in the delay period (right). (B) PPC spike-field coherence spectrum of the population of the FEF units with the LFP's recorded in the ACC across the alpha frequency range. Comparison between baseline and delay of contra-versive saccades (left), comparison between baseline and delay of ipsi-versive saccades (middle) and comparison between the contra- and ipsi-versive saccades in the delay period (right). Error bars denote SEM in all panels. *: $p < 0.05$, paired t-test.

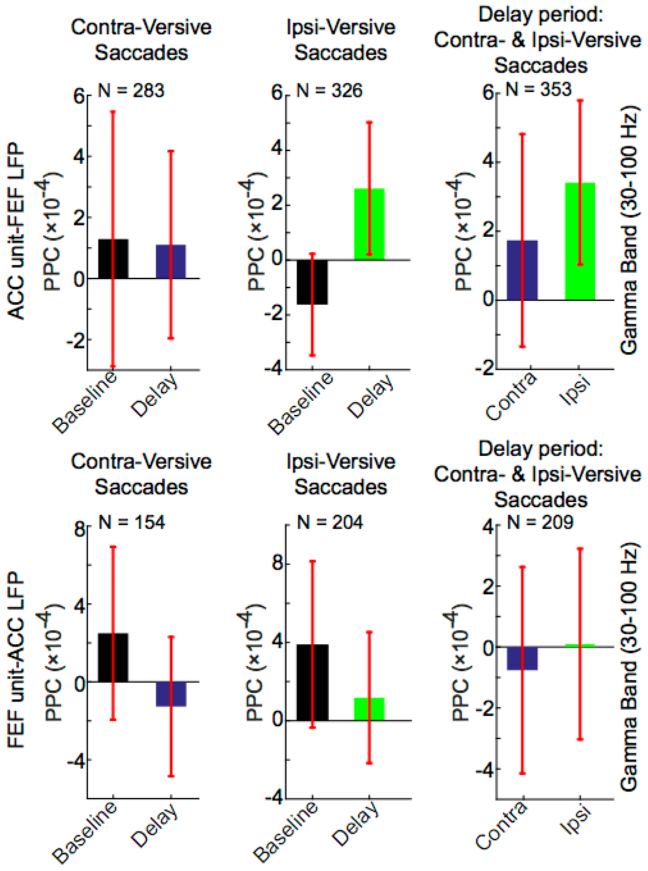

Supplementary Figure 6. Pairwise phase consistencies (PPCs) across gamma band. (A) PPC spike-field coherence spectrum of the population of the ACC units with the LFP's recorded in the FEF across the gamma frequency range. Comparison between baseline and delay of contra-versive saccades (left), comparison between baseline and delay of ipsi-versive saccades (middle) and comparison between the contra- and ipsi-versive saccades in the delay period (right). (B) PPC spike-field coherence spectrum of the population of the FEF units with the LFP's recorded in the ACC across the gamma frequency range. Comparison between baseline and delay of contra-versive saccades (left), comparison between baseline and delay of ipsi-versive saccades (middle) and comparison between the contra- and ipsi-versive saccades in the delay period (right). Error bars denote SEM in all panels.

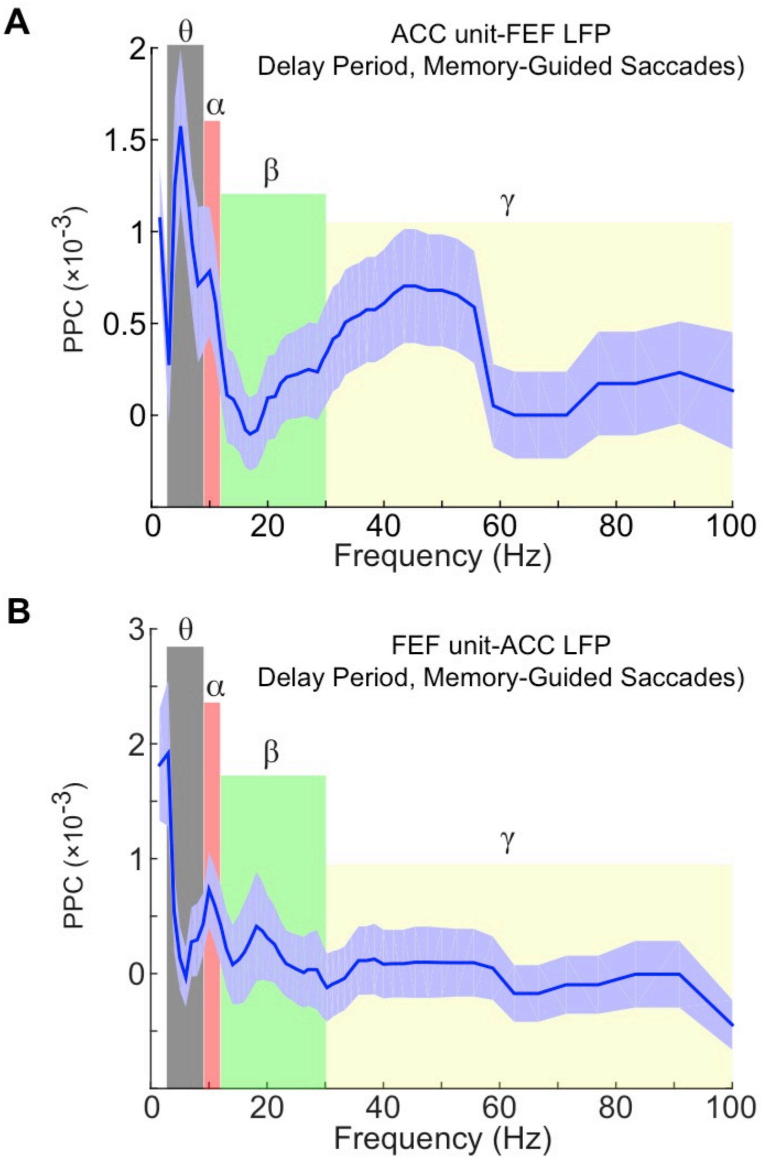

Supplementary Figure 7. Pairwise phase consistencies (PPCs) spectrum. (A) PPC spike-field coherence spectrum of the population of the ACC units with the LFP's recorded in the FEF across the frequency range of 1.5-100 Hz in the delay period of memory-guided saccade task. (B) PPC spike-field coherence spectrum of the population of the FEF units with the LFP's recorded in the ACC across the frequency range of 1.5-100 Hz in the delay period of memory-guided saccade task. In both panels, the peak spike-field coupling is observed in theta frequency range. Shades denote +/- SEM.

Supplementary References:

1. Van Essen DC, Glasser MF, Dierker DL, Harwell J. Cortical parcellations of the macaque monkey analyzed on surface-based atlases. *Cereb Cortex* **22**, 2227-2240 (2012).

2. Truccolo WA, Ding M, Knuth KH, Nakamura R, Bressler SL. Trial-to-trial variability of cortical evoked responses: implications for the analysis of functional connectivity. *Clinical neurophysiology : official journal of the International Federation of Clinical Neurophysiology* **113**, 206-226 (2002).
